# Site-Specific Fracture Healing: Comparison between Diaphysis and Metaphysis in the Mouse Long Bone

**DOI:** 10.3390/ijms22179299

**Published:** 2021-08-27

**Authors:** Satoshi Inoue, Jiro Takito, Masanori Nakamura

**Affiliations:** Department of Oral Anatomy and Developmental Biology, School of Dentistry, Showa University, 1-5-8 Hatanodai, Shinagawa-ku, Tokyo 142-8555, Japan; takito@dent.showa-u.ac.jp (J.T.); masanaka@dent.showa-u.ac.jp (M.N.)

**Keywords:** fracture healing, diaphysis, metaphysis, medullary callus, bone remodeling, estrogen, ovariectomy, *Hox* genes, skeletal stem cells

## Abstract

The process of fracture healing varies depending upon internal and external factors, such as the fracture site, mode of injury, and mechanical environment. This review focuses on site-specific fracture healing, particularly diaphyseal and metaphyseal healing in mouse long bones. Diaphyseal fractures heal by forming the periosteal and medullary callus, whereas metaphyseal fractures heal by forming the medullary callus. Bone healing in ovariectomized mice is accompanied by a decrease in the medullary callus formation both in the diaphysis and metaphysis. Administration of estrogen after fracture significantly recovers the decrease in diaphyseal healing but fails to recover the metaphyseal healing. Thus, the two bones show different osteogenic potentials after fracture in ovariectomized mice. This difference may be attributed to the heterogeneity of the skeletal stem cells (SSCs)/osteoblast progenitors of the two bones. The *Hox* genes that specify the patterning of the mammalian skeleton during embryogenesis are upregulated during the diaphyseal healing. *Hox* genes positively regulate the differentiation of osteoblasts from SSCs in vitro. During bone grafting, the SSCs in the donor’s bone express *Hox* with adaptability in the heterologous bone. These novel functions of the *Hox* genes are discussed herein with reference to the site-specificity of fracture healing.

## 1. Introduction

Bone is a mineralized connective tissue with multiple functions such as supporting the skeleton structure; producing new blood cells; shielding the internal organs, providing a scaffold for the muscles, ligaments, and tendons; and acting as a reservoir for minerals [1]. The bone remodeling cycle maintains the identity of each bone in the body. Bone remodeling occurs via the coordinated actions of the bone-forming osteoblasts, bone-degrading osteoclasts, and osteocytes that act as mechanosensors in the bone-remodeling compartment [2]. The differentiation and function of these cells are regulated by molecules such as growth factors and cytokines, as well as the mechanical environment. Furthermore, the remodeling cycle is consolidated by tightly coupled cell-cell interactions. The osteoblasts and osteocytes transmit a differentiation signal by presenting receptor activator of nuclear factor kappa-B ligand (RANKL) to osteoclast progenitors [3,4,5]. The bilateral signaling between the ligand ephrinB2 on osteoclasts and its receptor, EphB4, on the osteoblasts enhances osteoblast differentiation and inhibits osteoclast function, resulting in the phase transition from bone resorption to bone formation [6]. This remodeling cycle maintains the homeostasis of the bone interacting with the environment in the physiological state.

Bone injury damages the bone and interrupts the remodeling cycle. Fracture healing is a regenerative process that fills the discontinuity of the broken bone and returns the remodeling cycle. Incomplete healing, such as fracture nonunion, malunion, and delayed healing, has been a clinical problem [7]. Identification of the factors determining the processes of fracture healing may be useful for understanding the mechanism of regeneration and for better clinical treatment. Although fracture healing involves bone repair, reorganization of the blood vessel network [8], and remodeling of the bone marrow [9], this review focuses on the processes of bone repair. As remodeling is pivotal in bone homeostasis, the dynamic nature of remodeling appears to be a driving force for bone regeneration. Healing a bone fracture via the formation and degradation of bony callus is similar to bone remodeling [10]. There are two healing patterns: direct and indirect. Direct healing (also called intramembranous ossification) indicates the direct differentiation of bone-forming osteoblasts from skeletal stem cells (SSCs)/osteoblast progenitors (also called mesenchymal stem cells) [11]. Indirect healing (also called endochondral ossification) incorporates the stage of formation and degradation of the cartilaginous callus before osteoblast differentiation. The healing pattern varies with respect to the fracture site, mode of injury, and mechanical environment [12,13,14]. Traditionally, the mechanism of intramembranous and endochondral ossification in animals has been studied for healing fractures in the calvarium and diaphysis of long bones, respectively [14]. However, bone fractures in humans mainly occur in the metaphyseal regions of the long bone [15]. Several studies have indicated that metaphysis fractures heal via intramembranous ossification in rats [16], rabbits [17], and mice [18,19]. The difference in the healing patterns between the diaphysis and metaphysis of the long bones has not been well recognized. Recent studies in our lab have shown that the two bone regions heal via distinct patterns in a mouse fracture model [12,20]. In particular, the process of metaphyseal healing in ovariectomized (OVX) mice differs from that in normal mice. This review summarizes the differences in bone repair between the diaphysis and metaphysis in animals and discusses the origin of these differences.

## 2. Animal Models of Fracture Healing

**Figure 1:**
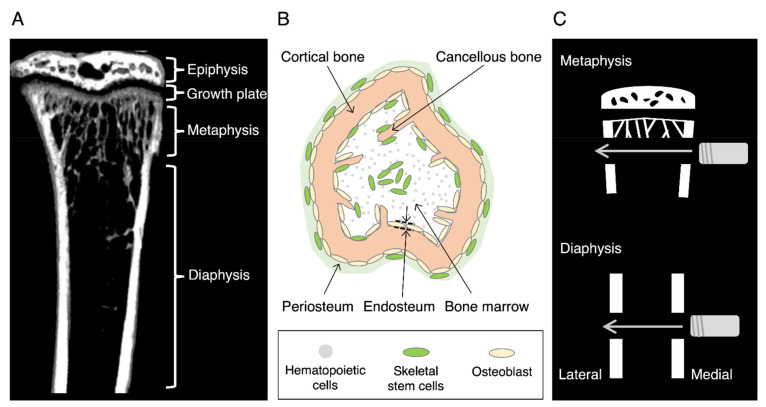
Mouse tibia and the fracture model used. (**A**) An image of the mouse tibia obtained through micro-computed tomography. (**B**) Schematic of a cross view of the mouse tibia. (**C**) A drill hole model for fracture healing. A drill hole is formed from medial to lateral through both sides of the cortical bones using a round bur.

A long bone consists of the epiphysis at both ends, diaphysis at the center, and metaphysis at the boundary of both sites [21] (Figure 1A). The diaphysis is composed of a thick cortical bone and a small amount of cancellous bone (also called trabecular or sponge), and the periosteum covers the outer surface of the cortical bone (Figure 1B). The bone marrow fills the medullary cavity. In contrast, the metaphysis consists of a thin cortical bone and a large amount of cancellous bone with a rich blood supply in the bone marrow. In children, a growth plate exists adjacent to the epiphysis and is responsible for the longitudinal growth of the bone. Rapid longitudinal growth often causes metaphysis fractures during puberty [22]. In the elderly, bone fractures occur primarily because of bone fragility owing to osteoporosis [23]. An increase in metaphysis fractures, such as the proximal femur and distal radius, has become a public health concern in developed countries. Researchers have devised various animal models to elucidate the mechanism of fracture healing for developing effective clinical care [24,25,26]. 

### 2.1. Closed Fracture Model

Since the pattern of fracture healing varies with age, sex, fracture site, and fracture severity, a suitable fracture model should be adopted to evaluate the mechanism of the specific healing process. The characteristics of various models devised so far have been reviewed elsewhere [25,26]. In short, there are two animal healing models: stable and unstable. The unstable model is accompanied by uncontrolled pain and fracture mobility, making it inappropriate for quantitative analysis. In a stable model, the injured site is stabilized by inserting a pin or a needle into the medullary cavity of the long bone. The closed fracture models in which the skin remains intact, heal via endochondral ossification. The cartilaginous callus appears at the periosteum region in these models, although the damage to the bone marrow by inserting a pin prevents the formation of the medullary callus. 

### 2.2. Open Fracture Model

In the open-fracture model, the skin is incised, and bone injury is produced by various methods. The injured site is often stabilized by the external fixators [25,26]. Rigid fixation allows the fracture to heal via intramembranous ossification. We adopted a drill hole model to compare the diaphysis and metaphysis healing (Figure 1C). Because of the partial bone defect, the fracture site was stable without fixation in this model. An aperture was formed in the diaphysis or metaphysis from the medial through both sides of the cortical bone using a round bur via open surgery. The surgery, however, required special care to preserve the intact skin, although the periosteum was broken at the lateral side of the cortical bone. The healing processes at the lateral side of the periosteum where the skin was intact were examined because the bone healing process in the periosteum region is sensitive to soft tissue injury. This model is therefore not completely fractured, unlike human fractures.

## 3. Fracture Healing in a Drill Hole Model

### 3.1. Diaphysis Healing

Diaphysis healing involves complex spatio-temporal processes with different mechanisms at specific locations. On the periosteal side, the cartilaginous and bony callus (periosteal callus) appear during fracture healing [10], whereas only the bony callus (medullary callus) emerges within the bone marrow cavity [12,27,28]. Therefore, here we separately describe the temporal events of diaphysis healing at three distinct locations.

#### 3.1.1. Endochondral Ossification at the Periosteal Side of the Cortical Bone

**Figure 2:**
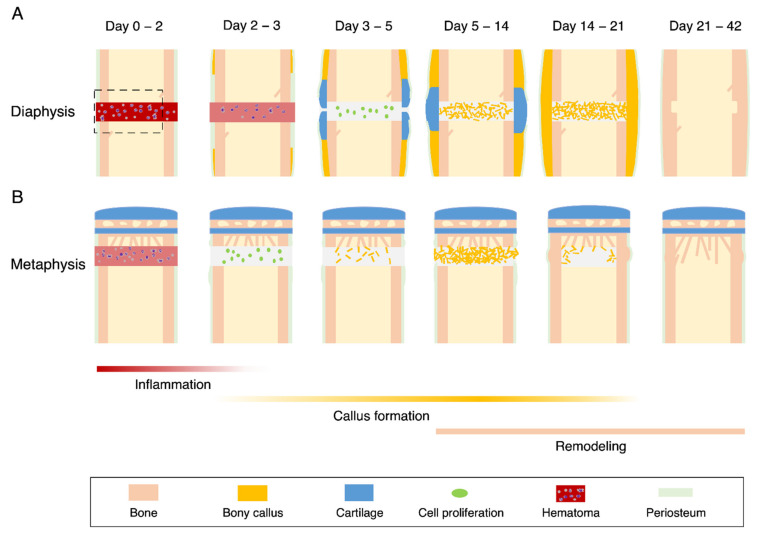
Schematic diagram of diaphysis and metaphysis healing in a mouse drill hole model. (**A**) Diaphysis fracture heals via the formation of the periosteal and medullary callus. (**B**) Metaphysis fracture heals via the formation of the medullary callus. The broken square indicates the region of interest for our analysis.

Fracture healing is divided into four histological stages on the periosteal side: inflammation, cartilaginous callus formation, bony callus formation, and remodeling [10] (Figure 2A). The first 3 days after fracture are characterized by hematoma formation [29] and subsequent inflammation reactions [30]. Fracture disrupts the local vascularization and soft tissues in the bone, resulting in hematoma formation. Immediately after the fracture, the platelets form a fibrin network that triggers hemostasis. In the inflammatory stage, platelets, neutrophils, macrophages, and lymphocytes are recruited to the fracture site in mice [30]. Neutrophils and macrophages remove dead cells and debris. The immune cells secrete pro-inflammatory cytokines, such as interleukin (IL)-1β, IL-6, and tumor necrosis factor α (TNF-α). They also release growth factors such as platelet-derived growth factor (PDGF), transforming growth factor-β (TGF-β), vascular endothelial growth factor (VEGF), and fibroblast growth factor (FGF) [31,32,33,34]. PDGF and TGF-β promote the recruitment and proliferation of SSCs at the fracture site [31,32]. FGF-2 activates the proliferation of osteoblast [33] and VEGF triggers angiogenesis [34]. On day 3, T and B cells are recruited to the fracture site [35]. They produce osteoprotegerin and RANKL to regulate osteoclast differentiation. The T cells secrete IL-17, which plays a role in osteoblastogenesis [36]. After day 4 (the cartilaginous callus formation stage), the SSCs differentiate into chondrocytes, forming a cartilaginous callus around the fracture site [37]. Accordingly, this stage is characterized by the high expression of *Sox9* and *type 2* and *type 10 collagen* mRNAs in mice and rats [12,37]. Periosteal cells appear to be the major source of progenitors for cartilaginous callus [27,37]. Other cells from different sources may also contribute to callus formation, including the bone marrow [38], vessel walls [39], surrounding muscles [40], and circulation [41]. SSCs within the periosteum show the higher regenerative potential than those in the bone marrow [42]. Notably, the cartilaginous callus did not belong to the primary cartilage. Embryologically, the cartilage is classified into primary and secondary cartilage [43]. The skeletal cartilage, such as the limb bud cartilage, is classified as the primary cartilage, which is formed by chondrocyte differentiation from the mesenchymal cell aggregates. On the contrary, cartilages in the maxillofacial region originate from the periosteum and are called secondary cartilages; they include the mandibular condylar cartilage, angular cartilage, and coronoid process. The cartilaginous callus formed during fracture healing was also classified as the secondary cartilage. After day 5, vascular invasion stimulates the replacement of the cartilaginous callus with the bony callus [8]. The bony callus formation stage is evident by the upregulation of *VEGF* mRNA at the ossification site in mice [44]. On day 14, the cartilaginous callus was completely replaced by a bony callus, while the bony callus was remodeled to the original bone architecture after day 21 by the coordinated action of the osteoclasts and osteoblasts [45]. 

Notably, the metaphyseal healing in a stable model (drill hole model) does not involve periosteal callus formation, as described below, although the mRNA expression of the chondrogenic markers (*Sox9* and *type 2 collagen*) is weakly upregulated in the periosteal cells [12]. In contrast, the metaphyseal healing in an unstable model [46] as well as a stable model in rats [47,48] forms a cartilaginous callus on the periosteum side. These results suggest that chondrogenesis from the periosteal cells depends on the mechanical environment during metaphyseal healing.

#### 3.1.2. Intramembranous Ossification at the Periosteal Side of the Cortical Bone

Thirty-six hours after the fracture, SSCs’ proliferation was detected at the periphery of the fracture site on the periosteal side in rats [49]. On day 3, the SSCs differentiated into osteoblasts that formed a bony callus at the end of the injured cortical bone. Ossification in this region is verified by the mRNA expression of *osteonectin*, *osteocalcin*, *alkaline phosphatase*, and *type 1 collagen* [49]. Inside the bony callus, endochondral ossification occurs, as described in Section 3.1.1. 

#### 3.1.3. Medullary Callus

During the healing of the diaphysis, bony callus appears in the marrow cavity in mice [12,27] and rats [9]. The bony callus is often called the medullary callus and/or endosteal callus. In this review, we differentiated the callus in the central bone marrow as a medullary callus and that near the cortical bone as the endosteal callus. On day 4 after fracture, the SSCs proliferate and differentiate into osteoblasts, which directly produce new bone in the bone marrow [12,27]. In parallel, the mRNA expression of *osterix*, *Runx-2*, and *type 1 collagen* was upregulated in the bone marrow [12]. On day 14, the medullary callus increased in size and filled the bone marrow of the fracture site. The callus gradually decreased in size and disappeared before the establishment of the bone union in the cortical bone.

### 3.2. Metaphyseal Healing

Previous studies have shown that metaphysis fractures in mice heal by the direct bone formation from the bone marrow without forming cartilaginous callus on the periosteal side [12]. The histological stages of metaphyseal healing differ from those of the diaphyseal healing [17,50]. We described the processes of metaphyseal healing in a drill hole model (Figure 2B) following the previously described classification [50]: inflammation, cell proliferation, bony callus, and bone remodeling.

#### 3.2.1. Medullary Callus

In the first stage of healing, hematoma formation and limited inflammation occurred 2 days after the fracture. The inflammation reaction of the metaphyseal healing is weaker than that of the diaphyseal healing in mice [20] and rabbits [17]. The reaction is distinguished by the early disappearance of neutrophils and an increase in the number of granulocytes [51]. In contrast, there is an increase in the number of lymphocytes on days 3 and 5 after fracture in the diaphyseal healing [51]. Cell proliferation starts earlier in metaphyseal healing than in diaphyseal healing due to the short-term inflammation (Figure 2B).

In the bony callus formation stage, the SSCs are recruited to the injured site and differentiate into osteoblasts. Osteoblasts form the medullary callus that fill the marrow cavity at the site of injury. This coincides with the expression of osteogenic markers, such as alkaline phosphatase, osteocalcin, and type I collagen. These markers appear earlier in the metaphyseal healing than in the diaphyseal healing [12].

In the lamellar bone formation stage, the medullary callus transforms into a bony callus with mature lamellar bone. The bony callus, formed in the bone marrow, is gradually resorbed by the osteoclasts, and the injured metaphysis is restored to the original architecture. The medullary callus in metaphyseal healing appears 5 days after the fracture and reaches the maximum size (volume) at day 7 in mice [12]. In contrast, the medullary callus in the diaphyseal healing occurs on day 7 and attains its maximum on day 14. In this stage, the region at which the osteoclasts appear overlaps with that of the medullary callus [20]. The maximum size of the callus in metaphyseal healing is equal to that in diaphyseal healing. The medullary callus in the metaphyseal healing gradually dissolves 21 days after fracture, whereas the callus in the diaphyseal healing disappears at day 42. 

#### 3.2.2. Endosteal Callus

The endosteum is a subcompartment of the bone marrow that lines the inner surface of the cortical bone (Figure 1B). The appearance of the endosteal callus appears to be rare because only a few studies have reported its existence [52,53]. It is impossible to distinguish the endosteal callus from the medullary callus during metaphyseal healing in normal and OVX mice. The large medullary callus might mask the endosteal callus. Alternatively, the formation of a medullary callus predominates over that of an endosteal callus during fracture healing. In contrast, the endosteal callus is evident during metaphyseal healing in estrogen-administered OVX mice, as described later, and appears to originate from the SSCs/osteoblast progenitors that reside in the endosteum [54].

## 4. Estrogen in Fracture Healing

### 4.1. OVX Mice

Although fractures may occur at various ages, osteoporotic patients are at a high risk of fracture [15,23]. In postmenopausal women, bone fragility occurs due to decreased levels of estrogen [23]. One of the goals of bone research is to prevent and treat osteoporosis. Hence, fracture healing has been studied in female animals whose ovaries are removed (ovariectomy) [55]. Although OVX animals do not precisely represent the symptoms of osteoporotic patients, they have some similarities with postmenopausal bone loss in humans. OVX mice exhibit significant and minor losses in the cancellous and cortical bones of the long bone, respectively [56].

### 4.2. Diaphyseal Healing in the OVX Mice

Previous studies have shown profound effects on diaphysis healing in mice [57,58,59,60,61,62]. At the inflammatory stage, ovariectomy increases the circulating inflammatory cytokines such as IL-6 [63], and midkine (Mdk) [61] in mice. Furthermore, an increase in the number of neutrophils at the site of injury [20] and adipocytes in the bone marrow is observed in OVX mice [64]. However, ovariectomy has little effect on the formation of cartilaginous callus in mice [57] and rats [65]. In contrast, ovariectomy decreases the size of the periosteal bony callus in mice [57] and rats [65] and medullary callus in mice [20,59], consistent with the decrease in the mRNA expression of *osteocalcin*, *type 1 collagen*, and *P1NP* in mice [59]. Substance P is a neuropeptide regulating angiogenesis and thereby bone metabolism and fracture healing [66]. Ovariectomy decreases the level of substance P, impairing angiogenesis during fracture healing [66]. In the late stage, the bone strength of the fracture site in the OVX mice was significantly lower than that in the controls, delaying the union [57,65].

### 4.3. Metaphyseal Healing in the OVX Mice

**Figure 3:**
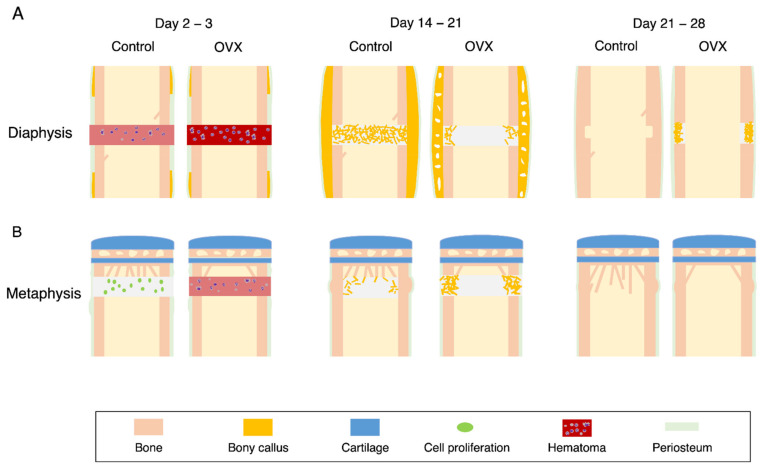
Effects of ovariectomy on the metaphyseal and diaphyseal healing in the mouse drill hole model. (**A**) Diaphyseal healing. (**B**) Metaphyseal healing. The fracture healing was compared at the inflammatory stage (**left**), callus formation stage (**center**), and remodeling stage (**right**).

Ovariectomy also deteriorates the metaphyseal healing in rats [47,67] and mice [20]. In mice, ovariectomy delays inflammation and is characterized by the late disappearance of neutrophils [20] (Figure 3). Additionally, ovariectomy decreases the formation of medullary callus, while it increases the endosteal callus formation. The decrease in the medullary callus appears to be compensated by an increase in the endosteal callus. The endosteal callus is formed by the osteoconduction from the existing cortical bone at the endosteal surface [48]. Overall, ovariectomy was found to delay the recovery of bone mineral density (BMD) at the injured site.

### 4.4. Effects of Estrogen Administration on Fracture Healing in the OVX Animals

The effects of estrogen on fracture healing in the OVX animals are summarized in Table 1. The subcutaneous injection of estrogen after bone injury partly rescues diaphyseal and metaphyseal healing in OVX mice [20]. Interestingly, estrogen significantly increased the amount of medullary callus in diaphyseal healing but caused no change in the medullary callus during metaphyseal healing, suggesting that the cells involved in medullary callus formation have varying sensitivities to estrogen between the diaphysis and metaphysis. A previous study reported that estrogen improves metaphyseal healing via an increase in the medullary callus in OVX rats [48]. In these experiments, the OVX rats had a significant amount of cancellous bone in the metaphysis, because they were fed estrogen for 10 weeks before bone injury. In addition, metaphyseal healing in this model is accompanied by the appearance of periosteal callus [47]. Therefore, the discrepancy may be attributed to the differences in experimental protocols. Unexpectedly, this discrepancy underlines the importance of pre-existing cancellous bone in the formation of medullary callus during metaphyseal healing.

In the late stage of fracture healing, osteoclasts resorb the bony callus and reshape the regenerated bone. The increased number of osteoclasts in the OVX animal bone is expected to shorten the duration of the remodeling stage. Consistent with this, preponderant osteoclasts appear at the periosteum region and persist longer in the OVX mice than in the controls [20]. In the future, quantitative estimation of the osteoclast activity at the late stage of fracture healing is required.

### 4.5. Phenotypes of Estrogen Receptor Alpha Knockout Mice

The comparison of fracture healing between the diaphysis and metaphysis indicates that the former is more severely affected by ovariectomy [20]. Here, we discuss this issue based on the phenotypes of the cortical and cancellous bones of the long bones of female mice with a cell-specific deletion of ERα [68]. Mice with deleted ERα in the osteoclasts via Ctsk-Cre [69] and LysM-Cre expression [70] exhibited attenuated bone mass in the cancellous bone but not in the cortical bone. These phenotypes are similar to those observed in the OVX mice, suggesting that estrogen regulates cancellous bone mass via osteoclastic bone resorption. However, deletion of ERα in the osteoblast progenitors via Prrx1-Cre and Sp7-Cre expression decreases the bone mass of the cortical bone due to decreased periosteal bone formation, without affecting the cancellous bone mass [71]. Therefore, estrogen plays a critical role in osteoblast differentiation in the periosteal region of the cortical bone. Do these results coincide with the observations during fracture healing in the OVX mice? Ovariectomy attenuates the formation of the periosteal bony callus during diaphyseal healing [57,65]. In metaphyseal healing, ovariectomy promotes the endosteal callus formation but inhibits the medullary callus formation, as described above. These observations support the notion that estrogen deficiency decreases the periosteal callus formation via osteoblastogenesis in diaphyseal healing and attenuates medullary callus formation via osteoclastogenesis in metaphyseal healing. Fracture healing involves bone regeneration; hence, osteoblasts might play a more important role than osteoclasts during healing. This probably explains the severe effect of ovariectomy on diaphyseal healing compared to metaphyseal healing. Therefore, facilitating osteoblastogenesis without inhibiting osteoclastogenesis may be more effective for fracture healing in osteoporosis patients.

## 5. Cells Involved in Fracture Healing

Fracture healing is an outcome of the complex actions of multiple cells at distinct stages. Both endochondral and intramembranous ossification processes involve stages of inflammation, bony callus formation, and remodeling. Here, we have summarized the roles of macrophages, osteoblasts, and osteoclasts at the respective stages.

### 5.1. Macrophages

Macrophages play a pivotal role in bone maintenance in the physiological state [72]. The Lys-M Cre/macrophage Fas-induced apoptosis (Mafia) mice (macrophage-depleted mice) at 3 months of age showed a significant decrease in the thickness and BMD of the cortical bone in the diaphysis and the number of cancellous bones and BMD in the metaphysis compared to the control, due to a deterioration in the proliferation of the osteoblast progenitors and the differentiation of the osteoblasts in the LysM Cre/Mafia mice. During fracture healing, the macrophages secrete various inflammatory cytokines, contributing to hematoma formation during the inflammation stage [10,30]. The secreted cytokines are also involved in the proliferation of SSCs, differentiation of osteoblasts, and differentiation of osteoclasts. The bone-resident macrophages (osteomas) are in direct contact with osteoblasts on the bone surfaces during diaphyseal healing in mice [73]. The in vivo macrophage depletion experiments using the transgenic Mafia mouse or clodronate liposome delivery demonstrated that the resident macrophages promote the formation of medullary callus in intramembranous ossification. Inflammatory macrophages are required for the formation of cartilaginous callus, and both inflammatory and resident macrophages are required for the formation of bony callus in the endochondral ossification during diaphyseal healing in a mouse flexible plate fracture gap model [74]. This is consistent with the finding that diaphysis healing in LysM Cre/Mafia mice exhibited reduced cartilaginous and bony callus in an osteotomy model stabilized with an intramedullary pin fracture model [75]. In the diaphyseal healing in a mouse intramedullary pin fracture model, a macrophage subset switch from M1 (inflammatory) to M2 (anti-inflammatory) is coupled with the transition from the cartilaginous callus to the woven bone callus in the endochondral ossification [76]. Thus, macrophages are involved not only in the cartilaginous and bony callus formation in the endochondral ossification but also in the bony callus formation during intramembranous ossification.

In the inflammatory stage, many cells assemble at the injured site and secrete pro-inflammatory and anti-inflammatory cytokines and growth factors, leading to complicated pharmacological interventions for fracture healing [77]. Antibiotics, anticoagulants, and non-steroidal anti-inflammatory drugs (NSAIDs) elicit negligible or negative effects on fracture healing. In a mouse drill hole and screw model, indomethacin and dexamethasone were reported to inhibit diaphyseal healing, whereas they showed minimal effects on the metaphyseal healing [18,19]. Therefore, these drugs may be useful in elucidating the mechanism of site-specific differences in fracture healing. 

### 5.2. Osteoblasts

A medullary callus appears in both diaphyseal and metaphyseal healing processes in a mouse drill hole model. Although the dynamics of the periosteal callus in endochondral ossification have been well elucidated, the dynamics of medullary callus have been elusive. Here, we summarize the current knowledge on the formation and disappearance of medullary callus.

The differentiation of SSCs into osteoblasts is the main mechanism of medullary callus formation. The lineage specification of SSCs is regulated by the specific transcription factors in response to the chemical, physical, and biological cues [78,79]. Although physical cues, including matrix stiffness [80], the micro-geometrical pattern of the matrix [81], and fluid shear stress [2] are known to determine the differentiation of osteoblasts from SSCs in vitro, the significance of these factors in fracture healing has been elusive. There exists an alternative route for osteoblast differentiation, that is, transdifferentiation of chondrocytes into the osteoblasts. Transdifferentiation occurs in the periosteal callus during fracture healing [82], but not in the endosteal and medullary callus. Bone graft healing experiments showed that SSCs at the periosteum, endosteum, and bone marrow form the bony callus at the respective site, indicating the importance of a site-specific environment [27]. The inverse orientation graft experiments further suggest the plasticity and priority of local SSCs for the formation of a prospective bony callus. Indeed, when the bone marrow or endosteum is removed before fracture, the amount of medullary callus decreases in non-stabilized fracture healing [9,27]. These results coincide with the idea that osteoblast progenitors that form the bony callus are derived from the proximal pool but not from the distant pool. In contrast, the transplanted SSCs are systemically transported to the site-specific niche at the fracture site and contribute to the formation of cartilaginous and bony callus in a stabilized mouse tibia fracture model [83]. The site-specific SSCs have been shown to have different properties. The mouse SSCs present on the endosteum have a stronger osteogenic potential than those in the central bone marrow [84]. The metaphysis with a rich cancellous bone harbors more SSCs than the diaphysis [84]. In addition, the metaphysis has more CD31^high^/endomucin^high^-positive vessels that contain abundant osteoprogenitor cells than those in the diaphysis [85]. The different sources of SSCs may independently contribute to the formation of the medullary callus. The differentiated osteoblasts at distinct sites exhibit different characteristics. The primary osteoblasts from rat calvaria and femur show differences in mineralization and gene expression in response to stimuli [86]. The importance of a geometrical factor for the behavior of medullary callus is shown during bone union in a rat osteotomy model [52]. When the fracture gap was 1 mm, the two medullary callus outside the gap formed endosteal bridging; however, when the gap was 5 mm, the medullary callus failed to form endosteal bridging, but closed the marrow cavities, resulting in bone nonunion. This indicates that the distance of the fracture gap determines the behavior of osteoblasts in the medullary callus. Curiously, the role of medullary callus in the intramembranous ossification has not been seriously pursued. Undoubtedly, a part of the medullary callus transforms to the cancellous bone at the late stage of fracture healing. The callus appears to confer stability to the unstable fracture site during healing. This idea is supported by the fact that the size of the medullary callus correlates with bone strength [87]. Future studies are needed to clearly define the role of the medullary callus during fracture healing.

Parathyroid hormone (PTH), approved for osteoporosis therapy, is expected to improve fracture healing. Abaloparatide, a PTH receptor agonist, increases callus size and callus bridging in a rat diaphysis femoral fracture model [88]. Teriparatide, human PTH (1-34), has positive or no effects on fracture healing rate and bone union [89,90]. The other group reported improved functional outcomes but not fracture healing rate [91]. Teriparatide enhances the osseous union after lumbar interbody fusion for osteoporosis-associated lumbar degenerative disorders [92]. Therefore, anabolic drugs appear to be useful in clinical applications. 

### 5.3. Osteoclasts

As described in Section 1, osteoclasts are indispensable for bone remodeling. Therefore, osteoclasts are also expected to function in the remodeling of the broken bones and bony callus during fracture healing. Indeed, both cartilaginous and bony callus formed after fracture completely degenerated before the completion of fracture healing. Antiresorptive drugs such as alendronate and denosumab (a RANKL inhibitor) delay the removal of cartilage and bony callus with a concomitant increase in mechanical stiffness during rat femur fracture healing [93]. However, alendronate has been reported to have no effect on the human distal radius fracture healing [94] or cause a slight delay in healing [95]. Similarly, denosumab does not affect human non-vertebral fracture healing [96], whereas it improves screw fixation in rat tibial fractures [97] and human pedicle screw fixation [98]. Therefore, antiresorptive drugs that effectively treat bone diseases such as osteoporosis are less effective against fracture healing.

The nature of osteoclasts in the diaphysis and metaphysis during fracture healing has not been explored. The site-specific heterogeneity of osteoclasts has been reported from the progenitors to the phenotypes of mature osteoclasts [99]. Future studies may reveal the site-specific heterogeneity of osteoclasts in response to the microenvironment and external factors during fracture healing.

## 6. *Hox* Code in Skeleton

### 6.1. Cortical Bone versus Cancellous Bone

The arguments so far raise the question of whether the difference in fracture healing between the diaphysis and metaphysis can be explained by the properties of cortical and cancellous bone. The cortical bone differs from the cancellous bone in many ways. First, the cortical bone has a higher BMD than cancellous bone in the radius and tibial diaphysis [100] and lumbar vertebrae in humans [101]. In mouse tibial metaphysis, the cortical BMD is approximately four-fold higher than that of the cancellous bone [102]. Second, there is a clear difference in the dynamics of the two bones. The cancellous bone has a two-fold higher bone turnover than the periosteal and intracortical regions of the cortical bone in the ilium of postmenopausal women [103]. Exceptionally, the endosteal region of cortical bone shows a higher turnover than cancellous bone. High bone turnover is a risk factor for fractures [103]. The adaptation of cortical bone to mechanical load differs from that of the cancellous bone [104]. The cortical bone adopts the load in a dose-dependent manner, whereas the cancellous bone is nearly insensitive to the load in a mouse tibial axial compression loading model. Lastly, both the cortical and cancellous bones in a long bone are generated by a less-understood mechanism downstream of primary ossification during embryogenesis [105]. The mechanism of intramembranous ossification in the calvaria may differ from that of metaphyseal healing. At present, precise knowledge of the intramembranous ossification at distinct sites during development remains elusive.

Apart from the issues on cortical versus cancellous bones, it is clear that the genetic code somehow specifies the formation of a given bone at a specified location during development. One such code is the *Hox* gene, a subset of homeobox-domain-containing transcription factors, originally found in the fruit fly *Drosophila* [106]. The mutation in the *Hox* gene causes homeotic transformation, formation of a given organ in an incorrect place. *Hox* genes are arranged collinearly in a cluster, and their expression spatiotemporally coincides with the formation of body segments along the anterior-posterior axis during *Drosophila* embryogenesis. Thus, *Hox* genes provide positional information of the body plan of an embryo but do not form a specific segment or organ.

### 6.2. Hox Genes in Skeleton

In humans, 39 *HOX* genes are found and arranged in four gene clusters: *HOXA*, *HOXB*, *HOXC*, and *HOXD* [107]. Because these genes have functional redundancy between the paralogous groups, it is difficult to define their function by activating or inactivating a single gene. Nonetheless, extensive studies using compound mutants have revealed that *Hox* genes work in specifying the skeleton of the vertebrate embryos during development. In mice, the axial vertebrae from cervical to caudal are patterned by Hox4 to Hox11. The limb is divided into three segments: stylopod (humerus, femur), zeugopod (radius/ulna, tibia/fibula), and an autopod (wrist/forepaw, ankle/hind paw). These segments are specified by Hox9, Hox10, Hox11, Hox12, and Hox13, respectively [108]. In the rib cage, the rib and sternum are derived from the somatic mesoderm and lateral plate mesoderm, respectively. Hox5, Hox6, and Hox9 specify the rib pattern from the cranial to caudal regions [109]. In contrast, the three *Hox* genes pattern the sternum in a non-linear manner. These results suggest that *Hox* genes are indispensable for the normal patterning of the mouse skeleton during development. Interestingly, *Hox* genes are expressed in the adult skeleton in the same pattern established during development [110], raising the possibility that *Hox* genes also function in the adult skeleton. This notion is demonstrated by examining the phenotypes of mice with conditionally deleted *Hox11* gene at the adult stage [111]. The *Hox11* deletion at the adult stage causes a transformation of the normal lamellar bone into an abnormal woven bone-like matrix of disorganized collagens in the cortical bone of the ulna, whereas it induces no change in the cortical bone of the humerus. The results suggest that *Hox* genes specify the global pattern of the mammalian skeleton during embryogenesis and also act as a factor in bone remodeling at a specific site in adults.

### 6.3. Hox Genes in Fracture Healing

*Hox* genes are also involved in fracture healing. The *Hoxa2* and *Hoxd9* mRNAs were upregulated throughout fracture healing (day 2 to day 21) in a rat femur-controlled fixed model [112]. Both the Hox proteins are expressed in the osteoprogenitors at the periosteum, fibrocartilage, and osteoblasts in the newly formed woven bone in the fracture callus. The expression of *Hox* genes during mouse femur diaphysis healing was studied using global transcriptional analysis [113]. The expression of *Hoxa3, Hoxa4, Hoxa10, Hoxb1, Hoxb13, Hoxc6, Hoxc10, Hoxd3*, and *Hoxd13* showed a biphasic peak at day 3 and day 14 or 21 after a fracture. *Hoxa1, Hoxa2, Hoxa4, Hoxa5, Hoxb3, Hoxb6, Hoxb9, Hoxc5, Hoxd3*, and *Hoxd9* were upregulated at the late stage (days 14 and 21 after fracture), whereas *Hoxa4, Hoxa11, Hoxb2, Hoxb8*, and *Hoxc8* are upregulated during healing. Surprisingly, many *Hox* genes are upregulated during diaphysis healing. Since the results appear to contradict the concept of region-specific expression of *Hox* genes in the skeleton, the significance of diverse expression of *Hox* genes in this study needs further examination.

The diaphysis healing in *Hox11*-deficient mice ulna shows reduced chondrogenesis and delayed ossification, resulting in the delayed bone union in an unstabilized model [110]. In contrast, the diaphysis healing in the *Hox11*-deficient mouse femurs showed no abnormalities. Hox11 is specifically expressed in SSCs at the periosteum of the adult ulna. The conditional deletion of *Hox11* in adult mice prevents the terminal differentiation of osteoblasts at the endosteal surface of the cortical bone of the ulna [111]. Furthermore, osteocytes cannot form the lacuno-canalicular network in the ulna of the *Hox11*-deleted mice. These results suggest that Hox11 functions in the adult bone in a region-specific manner. Hox11 positively regulates the differentiation of chondrocytes and osteoblasts but not the maintenance and proliferation of SSCs. *Hox11*-deficient SSCs show defects in chondrocyte and osteoblast differentiation in vitro [114]. Finally, Hox11-expressing cells are proposed to serve as region-specific SSCs throughout animal life [115].

Do region-specific SSCs also work in the heterologous place? Leucht et al. challenged this question [116]. All bones in the body originate from the neural crest or mesoderm during development. The interchangeability of the two cells was tested using heterotopic transplantation experiments. Periosteum prepared from the neural crest-derived mandible was grafted to the injury site of the mesoderm-derived tibia, and vice versa. In this assay, homotopic grafting was found to heal the injury of the tibia and mandible via intramembranous ossification. The tibial defect transplanted with mandible-derived periosteum healed via intramembranous ossification. The mandibular defect transplanted with the tibial periosteum healed via endochondral ossification. Lineage-tracing experiments confirmed that the newly formed cartilage and bone were derived from the transplanted cells. Thus, SSCs in the tibial periosteum differentiate into chondrocytes at the mandibular defect. The SSCs in the tibial periosteum express Hoxa11, whereas those in the mandibular periosteum are *Hox*-negative. The transplanted tibial cells maintained their Hoxa11-positive status in the *Hox*-negative mandibular environment. In contrast, the *Hox*-negative mandibular cells transplanted into the tibia began to express Hoxa11 7 days after transplantation, suggesting a change in the Hox status in response to the environment. The relationship between Hox expression and the fate of SSCs was examined among four different origins of SSCs [117]. The periosteum from the frontal bone, parietal bone, hyoid, and tibia contain neural crest-derived *Hox*-negative, mesoderm-derived *Hox*-negative, neural crest-derived *Hox*-positive, and mesoderm-derived *Hox*-positive SSCs, respectively. SSCs isolated from adult bones retain the embryonic Hox status. Hierarchical cluster analysis of the transcriptome indicated that transcriptional profiles of four SSCs were well separated into two clusters defined by the Hox expression status. In an in vivo scratch injury assay, the *Hox*-positive (hyoid and tibia) periosteum produces osteoblasts, whereas the *Hox*-negative (frontal and parietal) periosteum forms the chondrocytes and osteoblasts. In the in vitro differentiation assay, the *Hox*-negative periosteum showed higher potential for osteogenic differentiation, whereas the *Hox*-positive cells exhibited higher capability for chondrogenic and adipogenic differentiation. FACS analysis showed that the *Hox*-positive cells expressed more markers for primitive stem cells than the *Hox*-negative cells. These results suggest that Hox status, rather than developmental cell lineage, determines the fate of the SSCs. 

The regulatory mechanism of osteoblastogenesis by *Hox* genes has not yet been elucidated. In in vitro osteoblastogenesis, the *HoxA* cluster expression is regulated by epigenetic mechanisms, such as promoter methylation [118]. The microRNA-23a cluster regulates the *HoxA* cluster expression at various stages of osteoblast differentiation [119]. In contrast, bone morphogenetic protein 2 induces Hoxa10, which results in the activation of Runx2, a key osteogenic transcription factor [120]. Interestingly, Hoxa10 directly activates osteogenic genes, such as alkaline phosphatase and osteocalcin, independent of Runx2. In non-osteoblastic cells, *Hox* genes regulate the activity of genes such as Myb, Sox4 [121], β3 integrin [122], TGF-β2 [123], and FGF-2 [124]. The proteins encoded by these genes are known to be involved in osteoblast differentiation. Apart from the role of Hox in osteoblastogenesis, *Hox* genes are also involved in hematopoiesis [125] and angiogenesis [126]. Therefore, decoding the *Hox* code in the skeleton has prospects for understanding the principle of regeneration of bone fractures.

## 7. Perspectives

In this review, we summarized the differences in fracture repair between the diaphysis and metaphysis, and these differences may have clinical importance. In diaphyseal healing, a bony callus in the periosteal region is a clinical criterion [127], whereas metaphyseal healing is assessed by the medullary callus [128]. When a periosteal callus is formed, the assessment of the medullary callus becomes difficult because the medullary callus within the bone marrow overlaps with the periosteal callus. Accordingly, the role of the medullary callus appears to be underestimated in fracture healing. This review focused on the formation of medullary callus from the SSCs. In fracture healing, the SSCs originate from three distinct sites: the periosteum, endosteum, and bone marrow. SSCs from different sites have different osteogenic potentials, leading to the idea of region-specific SSCs. The heterogeneity of SSCs has been studied in terms of the developmental cell lineage and the environment, the so-called niche. Lineage-tracing experiments with immunofluorescence discriminate the three types of stromal progenitors at different stages of differentiation in the mouse bone marrow [54]. This review attempted to relate the *Hox* genes with the heterogeneity of SSCs. Although the significance of *Hox* genes in skeletogenesis is widely recognized, the role of *Hox* expression in the adult bone has been elusive until recently. Because fracture healing is a process of tissue regeneration, it is not surprising that the stimulus of fracture changes the expression of *Hox* genes and provides positional information to the fractured bone during regeneration. The positional cue specified by the *Hox* gene is effective on one bone or more during development. Such long-range effects cannot explain the heterogeneity of SSCs in the periosteum, endosteum, and bone marrow of the fracture site. In the future, a detailed analysis of the transcriptome of these SSCs will provide insights into the novel role of the *Hox* genes in fracture healing. Because the differentiation of SSCs is regulated by various physical and biochemical factors, it would be interesting to know the relationship between these factors and *Hox* expression. Moreover, elucidation of the mechanism of regulation of stemness of SSCs in vitro will be instrumental for the future clinical use of SSCs in cell therapy and regenerative medicine.

## Figures and Tables

**Table 1:** Changes in fracture healing in the ovariectomized animals.

	Diaphysis		Metaphysis	
Estrogen injection	−	+	−	+
Inflammation stage				
Neutrophils	↑ [20,60]	ND	↑ [20]	ND
Inflammatory cytokines	↑ [60,61]	ND	ND	ND
Callus formation stage				
Cartilaginous callus	→ [57,65]	↑ [57]	NA	NA
		→ [62]*		
Periosteal callus (bony)	↓ [57,59,65]	↑ [57,62]*	NA	NA
Medullary callus	↓ [59]	↑ [20]	↓ [20,47,48]	→ [20]
				↑ [47,48]*
Osteogenic markers	↓ [59]	ND	↓ [20]	ND
Remodeling stage				
Bone mineral density	↓ [59]	↑ [20]	↓ [20]	↑ [20]
Bone strength	↓ [57,65]	↑ [57]	↓ [48]	ND

Fracture healing was compared between the ovariectomized- and Sham-animals. Ovariectomy causes many changes in the events of fracture healing and delays the healing (Estrogen injection −). The estrogen injection to the ovariectomized animals after bone fracture partially recovers the delay (Estrogen injection +). The references are shown in parentheses. Abbreviations: ↑ increased; ↓ decreased; → no change; * estrogen was administered before the bone injury; ND, not determined; NA, not applicable.

## Data Availability

Not applicable.
